# Panel of human cell lines with human/mouse artificial chromosomes

**DOI:** 10.1038/s41598-022-06814-3

**Published:** 2022-02-22

**Authors:** Narumi Uno, Shuta Takata, Shinya Komoto, Hitomaru Miyamoto, Yuji Nakayama, Mitsuhiko Osaki, Ryota Mayuzumi, Natsumi Miyazaki, Chiaki Hando, Satoshi Abe, Tetsushi Sakuma, Takashi Yamamoto, Teruhiko Suzuki, Yoshihiro Nakajima, Mitsuo Oshimura, Kazuma Tomizuka, Yasuhiro Kazuki

**Affiliations:** 1 Division of Genome and Cellular Functions, Department of Molecular and Cellular Biology, Faculty of Medicine, School of Life Science, Tottori University, 86 Nishi-cho, Yonago, Tottori 683-8503 Japan; 2 Chromosome Engineering Research Center, Tottori University, 86 Nishi-cho, Yonago, Tottori 683-8503 Japan; 3 Laboratory of Bioengineering, Faculty of Life Sciences, Tokyo University of Pharmacy and Life Sciences, 1432-1 Horinouchi, Hachiohji, Tokyo 192-0392 Japan; 4 Division of Radioisotope Science, Research Initiative Center, Organization for Research Initiative and Promotion, Tottori University, 86 Nishi-cho, Yonago, Tottori 683-8503 Japan; 5 Division of Experimental Pathology, Department of Biomedical Sciences, Faculty of Medicine, Tottori University, Yonago, Tottori 683-8503 Japan; 6 Division of Integrated Sciences for Life, Graduate School of Integrated Sciences for Life, Hiroshima University, Higashi-Hiroshima, Hiroshima, 739-8526 Japan; 7 Stem Cell Project, Tokyo Metropolitan Institute of Medical Science, Kamikitazawa, Setagaya-ku, Tokyo 156-8506 Japan; 8 Health Research Institute, National Institute of Advanced Industrial Science and Technology (AIST), Takamatsu, Kagawa 761-0395 Japan

**Keywords:** Biological techniques, Biotechnology, Cell biology, Molecular biology, Stem cells

## Abstract

Human artificial chromosomes (HACs) and mouse artificial chromosomes (MACs) are non-integrating chromosomal gene delivery vectors for molecular biology research. Recently, microcell-mediated chromosome transfer (MMCT) of HACs/MACs has been achieved in various human cells that include human immortalised mesenchymal stem cells (hiMSCs) and human induced pluripotent stem cells (hiPSCs). However, the conventional strategy of gene introduction with HACs/MACs requires laborious and time-consuming stepwise isolation of clones for gene loading into HACs/MACs in donor cell lines (CHO and A9) and then transferring the HAC/MAC into cells via MMCT. To overcome these limitations and accelerate chromosome vector-based functional assays in human cells, we established various human cell lines (HEK293, HT1080, hiMSCs, and hiPSCs) with HACs/MACs that harbour a gene-loading site via MMCT. Model genes, such as tdTomato, TagBFP2, and ELuc, were introduced into these preprepared HAC/MAC-introduced cell lines via the Cre-loxP system or simultaneous insertion of multiple gene-loading vectors. The model genes on the HACs/MACs were stably expressed and the HACs/MACs were stably maintained in the cell lines. Thus, our strategy using this HAC/MAC-containing cell line panel has dramatically simplified and accelerated gene introduction via HACs/MACs.

## Introduction

**Figure 1:**
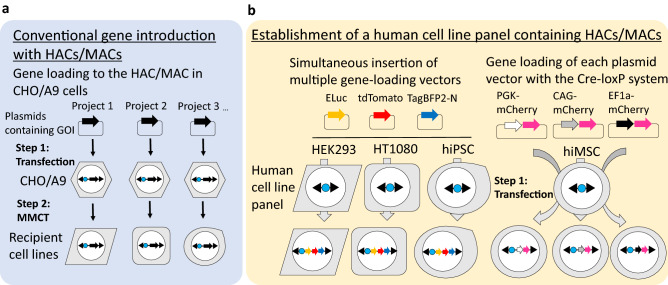
Schematic diagram of gene delivery via HACs/MACs. (**a**) Schematic diagram of the conventional gene-introducing strategy using HACs/MACs. In the conventional strategy, HACs/MACs for gene expression are constructed by transfection and insertion of the target gene(s) into the HAC/MAC in CHO or A9 cells that contain HACs/MACs as step 1. The constructed HACs/MACs are then introduced into target cells by MMCT as step 2. (**b**) Schematic diagram of the other gene-introducing strategy using the preprepared human cell panel with HACs/MACs. The HAC or MAC was transferred into representative human cell lines. The human cell line panel included HEK293 cells, HT1080 cells, human immortalised mesenchymal stem cells (hiMSCs), and human induced pluripotent stem cells (hiPSCs, 201B7). The human cell lines were directly available for gene loading into HACs/MACs with site-specific recombination. Simultaneous insertion of multiple gene-loading vectors into HEK293 cells, HT1080 cells, and hiPSCs (201B7) that contained HACs/MACs was tested by the SIM system. Each gene-loading vector of the SIM system contained Emerald luciferase (ELuc), a red fluorescent protein (tdTomato), or blue fluorescent protein (TagBFP2-N). hiMSCs that contained 21HAC2 were used to attempt insertion of three types of plasmid vectors that contained a red fluorescent protein (mCherry) driven by a different promoter with the Cre-loxP system. Each mCherry was expressed by a general constitutive promoter, e.g., PGK, CAG, or EF1α.

Human artificial chromosomes (HACs) and mouse artificial chromosome (MACs) have unique characteristics as vectors for gene delivery, which include stable and independent maintenance without disruption of the host genome and the capacity to carry numerous genes and megabase-sized genomic loci with physiological regulatory elements^1–4^. HAC/MAC technologies have been used for gene and cell therapies of Duchene muscular dystrophy^5–10^ and to generate trans-chromosomic (Tc) animals that include a mouse model of Down syndrome^11,12^ and humanised drug metabolism^13–18^. Furthermore, several types of HACs have been used in cancer research and drug development for cancer therapy^19–21^, centromere and telomere function elucidation^22^, a system of quantitatively tracking epigenetic memory in the field of synthetic biology^23^, and protein production^24^. To accelerate the gene loading of multiple genes into HACs/MACs, we have developed several systems for multiple gene insertions, such as the simultaneous or sequential integration of multiple gene-loading vectors (SIM) system^25,26^ via hypoxanthine–guanine phosphoribosyltransferase (HPRT)-deficient cells and drug screening with HAT, a multi-integrase (MI) system ^27–30^, and homologous recombination with clustered regularly interspaced short palindromic repeats (CRISPR)/CRISPR-associated protein 9 (Cas9)^31^. HACs/MACs are transferrable into desired cells by microcell-mediated chromosome transfer (MMCT)^32^. Although MMCT traditionally employs polyethylene glycol^33^, we developed a novel microcell membrane fusion method with the envelope proteins of measles virus (MV)^34,35^, amphotropic virus, and ecotropic virus^36^, which improved the transfer efficiency (1 × 10^−4^–1 × 10^−5^). However, specialised equipment and a laborious and time-consuming process are required for MMCT of HACs/MACs. This is because, in accordance with each experimental purpose, HACs/MACs with desired genes are constructed in CHO and A9 cells, and individually transferred to a target cell line via MMCT, and then clones are isolated, which contain the desired HACs/MACs (Fig. 1a). Therefore, we have previously employed mouse embryonic stem cells that contain a MAC with the MI system to facilitate the generation of Tc mice^37^. Under such circumstances, preprepared human cell lines that contain HACs/MACs will be a useful platform for simple and stable gene expression. To increase HAC/MAC applicability, we prepared HPRT-deficient human cell lines that contained HACs/MACs, which are applicable to gene loading by SIM and Cre-loxP systems in this study. The detailed structures of each HAC/MAC are shown in Supplementary Fig. S1. Hence, we report the generation of a human cell line panel for simple gene loading of genes of interest (GOI) using HACs/MACs (Fig. 1b). As representative human cell lines, we used HEK293, which is an immortalised human cell line, and HT1080, which is a cancer cell line, as well as a human immortalised mesenchymal stem cell (hiMSC) line^38,39^ and human induced pluripotent stem cell (hiPSC; 201B7) line^40^. We further attempted to adapt the Cre-loxP system (Supplementary Fig. S2a) and SIM system for multiple gene loadings (Supplementary Fig. S2b) in the generated human cell lines with HACs/MACs.

## Results and discussion

### Establishment of HAC/MAC-retaining human cell lines

Five types of mammalian artificial chromosomes, which included HACs and MACs, were used in this study (Supplementary Fig. S1). HACs were derived from human chromosome 21, such as 21HAC1 without EGFP and 21HAC2 with EGFP^41^, and MACs were derived from mouse chromosome 11, such as MAC2 without EGFP, MAC4 with EGFP ^37,42,43^, and MAC6 with EGFP. These HACs/MACs had a loxP site and partial HPRT gene as an acceptor site for the SIM system, which enabled simultaneous insertion of three circular plasmids. However, conventional gene introduction via HACs/MACs requires transfer of the HACs/MACs with desired gene(s) by MMCT into target cells and there is a major technical difficulty. In this study, we established a human somatic/stem cell line panel (HEK293 cell, HT1080 cell, hiMSC, and hiPSC lines) that contained HACs/MACs (Fig. 1) (Table 1).

**Table 1:** List of human cell lines that contained HACs/MACs.

Cell line	Component	EGFP	Drug resistance
HEK293	21HAC1	Absent	HygR, PuroR, HATS, and GancS
	21HAC2	Present	HygR, PuroR, BSR, HATS, and GancS
	MAC2	Absent	HygR, PuroR, and HATS
	MAC4	Present	HygR, PuroR, and HATS
HT1080	21HAC2	Present	HygR, PuroR, BSR, HATS, and GancS
	MAC4	Present	HygR, PuroR, and HATS
hiMSC	21HAC2	Present	HygR, PuroR, BSR, HATS, and GancS
hiPSC	MAC6 (ΔNeoR)	Present	PuroR and HATS

Cell lines, HACs/MACs, EGFP marker, and drug resistance are summarised. Regarding drug resistance, R indicates cells resistant to antibiotics, i.e., *Hyg*  hygromycin, *Puro* puromycin, *BS* blasticidin S, and S indicates that cells are selectable by drugs, i.e., *HAT* hypoxanthine-aminopterin-thymidine medium and *Ganc* ganciclovir.

**Figure 2:**
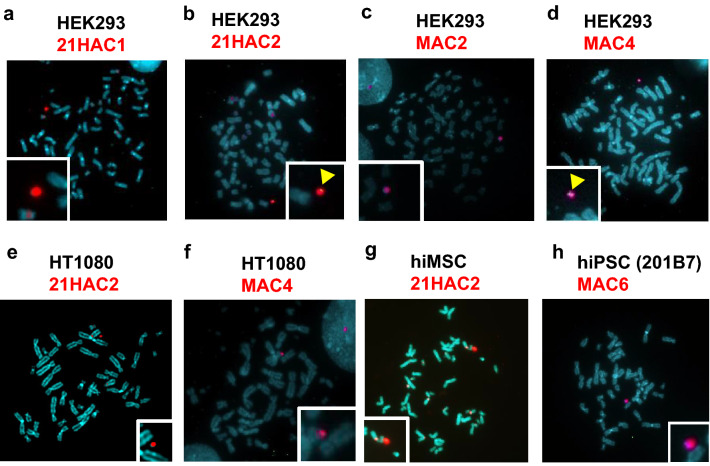
Representative images of FISH analyses of HEK293 cells, HT1080 cells, hiMSCs, and iPSCs that contained each HAC/MAC. (**a**) HEK293 cells that contained 21HAC1. Red: alpha satellite probe (p11-4) staining the centromere of Chr.13, 21, 21HAC1. (**b**) HEK293 cells that contained 21HAC2. Red: alpha satellite probe (p11-4) staining the centromere of Chr.13, 21 and 21HAC2; green: pCX-EGFP. (**c**) HEK293 cells that contained MAC2. Red: mouse cot-1 staining MAC2. (**d**) HEK293 cells that contained MAC4. Red: mouse cot-1 staining MAC4. (**e**) HT1080 cells that contained 21HAC2. Red: alpha satellite probe (p11-4) staining the centromere of Chr.13, 21, 21HAC1. (**f**) HT1080 cells that contained MAC4. Red: mouse cot-1 staining MAC4. (**g**) hiMSCs that contained 21HAC2. Red: alpha satellite probe (p11-4) staining the centromere of Chr.13, 21 and 21HAC2. (**h**) hiPSCs (201B7) that contained MAC6. Red; mouse cot-1 staining MAC6. Inset: enlarged images of each HAC or MAC.

Then, plasmid vector(s) with a GOI were inserted into the HAC/MAC via the SIM system for HEK293 cells, HT1080 cells, and hiPSCs or the Cre-loxP system for hiMSCs. Fluorescence in situ hybridisation (FISH) analyses revealed that a single additional HAC or MAC was maintained independently from the host chromosome in each somatic/stem cell line. Specifically, HEK293 cells contained 21HAC1 (Fig. 2a), 21HAC2 (Fig. 2b), MAC2 (Fig. 2c), or MAC4 (Fig. 2d), HT1080 cells contained 21HAC2 (Fig. 2e) or MAC4 (Fig. 2f), hiMSCs contained 21HAC2 (Fig. 2g), and hiPSCs (201B7) contained MAC6 (Fig. 2h). A summary of the cell line panel is described in Table 1. Regarding hiPSCs (201B7) with MAC6, the Neo resistance gene on MAC6 was disrupted (MAC6-ΔNeoR) for further gene insertion and drug selection. Although promoters for overexpression of transgenes often undergo gene silencing in human pluripotent stem cells^44^, HACs/MACs maintained the desired gene expression level in long-term cell culture, which differed from gene transduction with plasmid DNA via random insertion. Therefore, the insertion of the NeoR gene driven by the PGK promoter on MAC6 would be applicable to obtain a clone with an inserted circular plasmid vector for drug selection of hiPSCs. Thus, the NeoR gene on MAC6 in hiPSCs was knocked out to establish 201B7/MAC6-ΔNeoR cells (Supplementary Fig. S1e).

### Demonstration of gene loading with three vectors by simultaneous introduction using the SIM system into HACs/MACs in HEK293 and HT1080 cells

**Figure 3:**
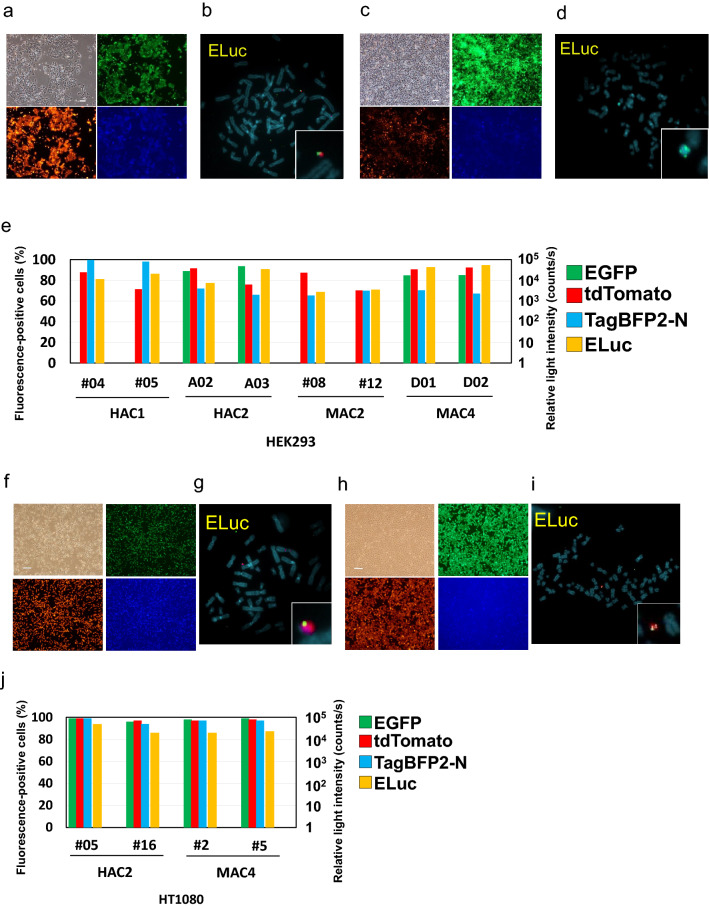
Analyses of HEK293 and HT1080 cells that contained HACs/MACs that harboured tdTomato, BFP, and ELuc via the SIM system. (**a**) Representative images of fluorescent protein expression in HEK293 cells that contained 21HAC2 inserted with tdTomato, BFP, and ELuc via the SIM system. Brightfield (top, left), tdTomato (Bottom, left), EGFP (top, right), and BFP (bottom, right). Bar = 200 µm. (**b**) Representative image of FISH analysis of HEK293 cells that contained 21HAC2 and the three plasmids. Red: alpha satellite probe (p11-4) staining the centromere of Chr.13, 21 and 21HAC2; green: pBG2-V0b-ins-ELuc-ins. (**c**) Representative images of fluorescent protein expression in HEK293 cells that contained MAC4 inserted with tdTomato, BFP, and ELuc via the SIM system. Bar = 200 µm. (**d**) Representative image of FISH analysis of HEK293 cells that contained 21HAC2 and the three plasmids. Green: pBG2-V0b-ins-ELuc-ins. (**e**) Two Y-axis graph showing the results of FCM analysis with 10,000 cells and luciferase assays of two clones of HEK293 cells that contained 21HAC1, 21HAC2, MAC2, and MAC4 inserted with the three plasmids. Left Y-axis shows the ratio of cells expressing each fluorescent protein in FCM analysis. Right Y-axis shows the photon counts (counts/second) in luciferase assays. Green, red, and blue bars show the ratio of cells expressing each fluorescent protein, and the yellow bar shows the photon counts of the luciferase assay of the clone. Two representative clones are shown for each experiment. (**f**) Representative images of fluorescent protein expression in HT1080 cells that contained 21HAC2 inserted with tdTomato, BFP, and ELuc via the SIM system. Bar = 200 µm. (**g**) Representative image of FISH analysis of HT1080 cells that contained 21HAC2 and the three plasmids. Red: alpha satellite probe (p11-4) staining the centromere of Chr.13, 21 and 21HAC2; green: pBG2-V0b-ins-ELuc-ins. (**h**) Representative images of fluorescent protein expression in HT1080 cells that contained MAC4 inserted with tdTomato, BFP, and ELuc via the SIM system. Bar = 200 µm. (**i**) Representative image of FISH analysis of HT1080 cells that contained MAC4 and the three plasmids. Red: mouse Cot-1 staining MAC4; green: pBG2-V0b-ins-ELuc-ins. (**j**) Two Y-axis graph showing the results of FCM analysis and luciferase assays of two clones of HT1080 cells that contained 21HAC2 and MAC4 inserted with the three plasmids. Two representative clones are shown for each experiment.

We demonstrated that somatic/stem cell lines that contained HACs/MACs accepted three plasmid vectors by simultaneous introduction using the SIM system (Supplementary Fig. S2b). As model genes for demonstration, a luminescent protein, Emerald luciferase (ELuc), and two fluorescent proteins, tdTomato and TagBFP2-N, were selected (hereafter collectively called ElTB). Specifically, we evaluated HEK293 cells that contained 21HAC1, 21HAC2, MAC2, or MAC4, HT1080 cells that contained 21HAC2 or MAC4, and hiPSCs that contained MAC6-ΔNeoR by introducing the three vectors with each model gene using the SIM system. Numerous drug-resistant clones were observed and an arbitrary number of clones were analysed by PCR to detect the correct insertion of the plasmid into the HAC/MAC. A summary of the PCR analysis is shown in Supplementary Table S1. The obtained drug-resistant clones were analysed by fluorescence microscopy, flow cytometry (FCM), and luciferase activity assays. These assays showed fluorescent proteins and luciferase expression in each cell line of representative HEK293 and HT1080 clones as expected. The results of HEK293/21HAC2-ElTB cells are shown in Fig. 3a,b,e, HEK293/MAC4-ElTB cells are shown in Fig. 3c–e, HT1080/21HAC2-ElTB cells are shown in Fig. 3f,g,j, and HT1080/MAC4-ElTB cells are shown in Fig. 3h–j. Representative results of the expression frequency of the introduced fluorescent protein gene and luciferase gene expression level were similar for each parental clone used for gene transfer. Therefore, to further expand the cell line panel, clones that show excellent gene expression should be selected. FISH analyses showed stable maintenance of HACs/MACs that contained the transgenes independently from host chromosomes. Fluorescence imaging of HEK293/21HAC1-ElTB and HEK293/MAC2-ElTB cells also showed expression of tdTomato and TagBFP2-N (Supplementary Fig. S3a,b). FISH analyses were also performed in HEK293/21HAC1 and HEK293/MAC2 cells that contained the vectors using the ELuc plasmid probe. The results showed that one HAC/MAC was maintained in each cell and that the transfected plasmid was inserted into the HAC/MAC as expected (Supplementary Fig. S3c,d). These results showed that multiple gene loadings into the HAC/MAC via the SIM system was achieved in our established human cell line panel (Fig. 2a–f), which enables seamless application of our HAC/MAC technology for gene function assays in human cells in the future.

### Characterisation of hiMSCs that contain HAC and demonstration of gene loading by the Cre-loxP system

**Figure 4:**
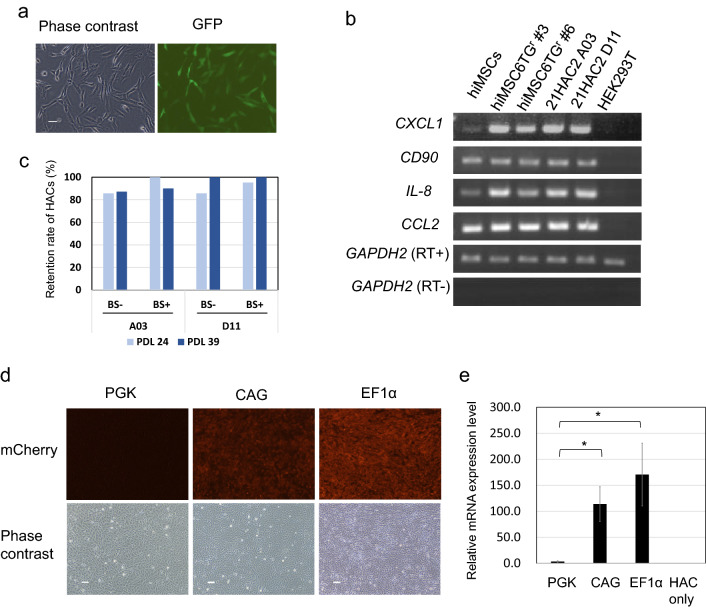
Characterisation of human immortalised mesenchymal stem cells (hiMSCs) that contained 21HAC2 and hiMSC/21HAC2 with the mCherry expression vector introduced by the Cre-loxP system. (**a**) Representative images of fluorescent protein expression in hiMSCs that contained 21HAC2. The left panel shows phase contrast and the right panel shows EGFP expression. Bar = 100 µm. (**b**) RT-PCR analysis of various MSC markers in hiMSCs that contained 21HAC2. The original gel images of the RT-PCR are provided in Supplementary Fig. S4. (**c**) Stability of 21HAC in each hiMSC clone (A03 and D11) after long-term cell culture with or without blasticidin S (BS). The measurement was performed at PDLs of 24 and 39. Bright blue bars show the retention ratio of 21HAC2 at PDL 24. Dark blue bars show the retention ratio at PDL 39 (**d**) Representative images of fluorescent protein expression in hiMSCs that contained 21HAC2. The left panel shows mCherry fluorescence under the control of the PGK promoter, the middle panel shows that under the control of the CAG promoter, and the right panel shows that under the control of the EF1α promoter. Bar = 200 µm. (**e**) Relative mRNA expression levels under the control of PGK, CAG, and EF1α promoters (n = 10) (*P < 0.01). *P*-values were calculated by the Student’s *t*-test.

hiMSCs with a transferred 21HAC2 (Fig. 2g), which were clones A03 and D11, stably expressed EGFP (Fig. 4a). Various MSC markers were also analysed by RT-PCR in these clones (Fig. 4b). Expression level of CXCL1 and IL8 is higher in resultant clones than in parental hiMSCs. CD90 is a typical MSC marker and the others including CXCL1 and IL8 are linked to therapeutic functionality. For the recipient hiMSC cell lines employed for HAC introduction, 6-thioguanine (6TG)-resistant clones (#3 and #6) were used to obtain HAT-resistant cell lines by HPRT gene reconstruction. The 21HAC2-carrying cell lines (A03 and D11) showed comparable or higher expression levels of the various MSC markers compared with the original hiMSC cell line. These results indicated that the hiMSC clones that contained 21HAC2 maintained the characteristics of MSCs (Fig. 4b). To evaluate the HAC retention ratio after long-term cell culture with or without an antibiotic (blasticidin; Bsd), FISH analysis was performed and results showed stable maintenance of the HAC at population doubling levels (PDLs) of 24 and 39, even without Bsd (Fig. 4c). These results indicated that the two hiMSC clones that contained 21HAC2 (hiMSC/21HAC2 A03 and D11) could be used as a platform for gene loading^38^. We validated whether 21HAC2 functioned as a safe harbour for gene expression in these established clones. As an example of functional analysis, we attempted to evaluate the expression level of the transgene promoted by three types of constitutive promoters (PGK, EF1α, and CAG) on 21HAC2 in hiMSCs with a defined (single in this study) copy number. Among the drug-resistant clones obtained by transfection with a plasmid that carried each promoter, 10 clones were picked up in order of the fluorescence intensity of mCherry under a fluorescence microscope and used for subsequent analysis to measure each promoter activity. Fluorescence imaging of mCherry-expressing cells with each promoter indicated that CAG and EF1α promoter activities were higher than the PGK promoter activity (Fig. 4d). qRT-PCR of the mRNA expression level of mCherry also indicated that CAG and EF1 promoter activities were higher at 22.4-fold (CAG) and 40.5-fold (EF1α) compared with the PGK promoter activity (n = 10) (*P* < 0.01) (Fig. 4e). There was no significant difference between the activities of CAG and EF1α promoters. These results supported a previous study that compared promoter activity in MSCs with a viral vector system for gene expression^45^. Because the Cre-loxP system had the same adaptor as the SIM system in 21HAC2, the SIM system would be applicable to hiMSCs/21HAC2. These results showed that hiMSCs that contained 21HAC2 were applicable to gene loading and functional analyses.

### Characterisation of hiPSCs that contained MAC and demonstration of gene loading by the SIM system

**Figure 5:**
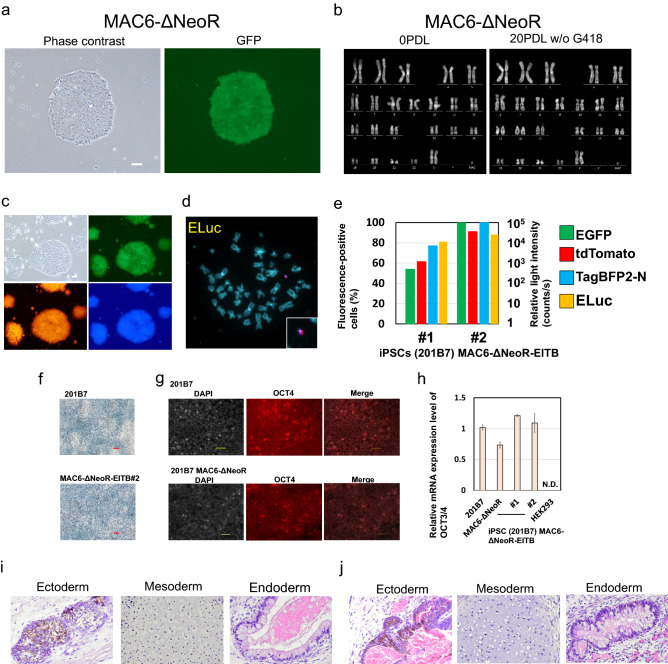
Characterisation of hiPSCs (201B7) that contained MAC6-ΔNeoR and 201B7/MAC6-ΔNeoR inserted with the three vectors by the SIM system. (**a**) Representative images of fluorescent protein expression in 201B7/MAC6-ΔNeoR cells. The left panel shows phase contrast and the right panel shows EGFP expression. Bar = 200 µm. (**b**) Karyotypes of hiPSCs (201B7)/MAC6-ΔNeoR during establishment (left, PDL 0) and in long-term cell culture (right, PDL 20). (**c**) Representative images of fluorescent protein expression in 201B7/MAC6-ΔNeoR cells inserted with tdTomato, BFP, and ELuc via the SIM system. Bar = 200 µm. (**d**) Representative image of FISH analysis of 201B7/MAC6-ΔNeoR cells with the three plasmids. Red: mouse cot-1 staining MAC4; green: pBG2-V0b-ins-ELuc-ins. (**e**) Two Y-axis graph showing the results of FCM analysis and luciferase assays of two clones of 201B7/MAC6-ΔNeoR cells inserted with the three plasmids. (**f**) Representative images of alkaline phosphatase staining of 201B7 and 201B7/MAC6-ΔNeoR-ElTB#2 cells. Bar = 50 µm. Alkaline phosphatase activity was detected by deposition of purple pigment. (**g**) Representative image of immunofluorescence staining for OCT3/4 in 201B7 and 201B7/MAC6-ΔNeoR cells. Bar = 50 µm. (**h**) Relative mRNA expression levels of OCT 3/4 in each cell line or clone. (n = 3). N.D. means not detected. (**i**) Teratoma formation analysis of 201B7/MAC6-ΔNeoR cells. Left, middle, and right panels show ectodermal, mesodermal, and endodermal tissues, respectively. (**j**) Teratoma formation analysis of 201B7/MAC6-ΔNeoR-ElTB#2. Left, middle, and right panels show ectodermal, mesodermal, and endodermal tissues, respectively.

hiPSCs (201B7) that contained MAC6-ΔNeoR expressed EGFP (Fig. 5a). We performed long-term cell culture without an antibiotic (G418) and quinacrine-Hoechst karyotyping to evaluate the HAC retention ratio. Karyotyping of hiPSCs showed an ideal karyotype that included a MAC, 47, XX, + MAC (Fig. 5b, left panel). The long-term culture of hiPSCs revealed that the karyotype and MAC were stable at a PDL of 20 (Fig. 5b, right panel). Next, we attempted gene loading with the SIM system into 201B7/MAC6-ΔNeoR cells. The obtained hiPSC clone [(201B7)/MAC6-ΔNeoR-ElTB] expressed tdTomato, TagBFP2-N, and EGFP (Fig. 5c). FISH analysis showed that the transgenes were integrated into the MAC and that the MAC was independently maintained in the hiPSCs (Fig. 5d). The expression level of each fluorescent marker and ELuc in 201B7/MAC6-ΔNeoR-ElTB cells was evaluated by FCM analysis and luciferase assays, respectively (Fig. 5e). Expression of all introduced genes was detectable among the representative two clones. Furthermore, 201B7/MAC6-ΔNeoR-ElTB cells showed a normal karyotype and stable maintenance of the MAC as well as 201B7/MAC6-ΔNeoR. In vitro, the expression of pluripotency markers was verified. The iPS clones 201B7/MAC6-ΔNeoR-ElTB showed activity of alkaline phosphatase and expression of OCT 3/4 as well as 201B7 (Fig. 5f,g). Quantitative RT-PCR analysis confirmed expression of OCT 3/4 in each clone (Fig. 5h). To verify the pluripotency of these iPSC lines, we tested their ability to differentiate into all three germ layers in vivo using the teratoma method. 201B7/MAC6-ΔNeoR and 201B7/MAC6-ΔNeoR-ElTB cells showed maintenance of pluripotency to differentiate into the three germ layers after gene loading and cloning (Fig. 5i,j). These results showed that 201B7/MAC6-ΔNeoR cells were applicable to gene loading with MAC technology.

## Conclusion

We generated a novel human cell line panel with HEK293 cell, HT1080 cell, hiMSC, and hiPSC (201B7) lines that contained HACs/MACs, which enabled rapid and precise insertion of GOIs at a defined site on HACs/MACs by a simple transfection method. The GOIs were stably expressed in each cell line, which indicated that the integration site can act as a “safe harbour” to support transgene expression. Thus, our new preprepared cell panel with HACs/MACs may dramatically simplify the construction of HACs/MACs with desired genes and constructed HACs/MACs can be used immediately and directly for functional analyses of genes in desired cell lines.

## Material and methods

### Ethics statement

This study was approved by the Institutional Animal Care and Use Committee of Tottori University (Permit numbers: 20-Y-14, 17-Y-27, and 16-Y-19) and the Recombinant DNA Experiment Safety Committee of Tottori University to perform recombinant DNA experiments. All experiments were carried out in compliance with the ARRIVE guidelines. All methods were performed in accordance with the relevant guidelines and regulations.

### Cell culture

CHO cells derived from a HPRT-deficient cell line (JCRB0218) (NIBIOHN, Osaka, Japan), which contained 21HAC1^41^, MAC2^43^, or MAC4^46^, were cultured in Ham’s F-12 medium (FUJIFILM Wako, Osaka, Japan) with 10% FBS, 1% penicillin/streptomycin (FUJIFILM Wako), and 800 µg/mL hygromycin B (FUJIFILM Wako). CHO cells that contained 21HAC2 were cultured in Ham’s F-12 medium with blasticidin S (FUJIFILM Wako). HEK293 cells were purchased from the ATCC (ATCC® CRL-1573™) and cultured in Eagle’s minimum essential medium (Sigma-Aldrich, St. Louis, MO, USA) with 10% fetal bovine serum (FBS, Biowest, Vieux Bourg, Nuaillé, France), 1% non-essential amino acids (Sigma-Aldrich), 1% L-glutamine (Sigma-Aldrich), and 1% penicillin/streptomycin (FUJIFILM Wako). HT1080 cells^47^ obtained from the ATCC (ATCC® CCL-121™) were cultured in Dulbecco’s modified Eagle’s medium (FUJIFILM Wako) with 10% FBS and 1% penicillin/streptomycin. The human immortalised mesenchymal stem cell (hiMSC)^39^ line was kindly provided by Dr. J. Toguchida and was cultured in Dulbecco’s modified Eagle’s medium (FUJIFILM Wako) with 10% FBS and 1% penicillin/streptomycin. HEK293 clones that contained each HAC/MAC were selected with 200 µg/mL hygromycin B (FUJIFILM Wako). HT1080 cell and hiMSC clones that contained HAC2 were selected with 8 and 4 µg/mL blasticidin S, respectively. HT1080 clones that contained MAC2 were selected with 200 µg/mL hygromycin B. For drug selection after transfection using the SIM system, HACs/MACs expressed the HPRT gene for hypoxanthine-aminopterin-thymidine (HAT) resistance following gene insertion. HEK293 and HT1080 clones were selected after transfection in HAT medium (Sigma-Aldrich). hiPSC cell line 201B7 (HPS0063) was provided by the RIKEN BRC and cultured in StemFit AK02N (Takara Bio, Kusatsu, Japan) with Laminin-511 (Nippi, Adachiku, Japan). hiPSCs that contained MAC6 with a neomycin resistance gene were selected with 90 µg/mL G418.

### Microcell-mediated chromosome transfer

To prepare microcells that contained 21HAC1, MAC2, or MAC4, 1 × 10^7^ chromosome donor CHO cells that contained each artificial chromosome were cotransfected with 12 μg pTNH6-H-αCD9 for HEK293 cells and hiPSCs or pTNH6-H for HT1080 cells and 12 μg pCAG-T7-F for each recipient cell line by Lipofectamine 2000 (Thermo Fisher Scientific, Waltham, MA, USA) in accordance with the manufacturer’s instructions^26,34^. CHO 4H6.1 M cells stably expressed MV-H and F, which provided microcells that contained 21HAC2 as reported previously^35^. Twelve flasks of CHO cells were prepared and micronuclei were induced by treatment with 0.1 µg/mL colcemid. The detailed MMCT protocol has been described previously^26^. The collected microcells were cocultured and fused with 2 × 10^6^ cells of each recipient cell line for 24 h in a 6-cm dish (Corning, Corning, NY, USA). Then, the fused recipient cells were subcultured into three 10-cm dishes. Drug selection was started with optimal selectable antibiotics after a further 24 h of incubation. After 14–21 days, drug-resistant colonies were picked up and expanded for the following analyses.

### Plasmid construction

To construct pBG-V0b1-ins-ELuc-ins, an EcoRV-digested fragment, which included an ELuc expression unit from CAG-ELuc, was ligated into pBG-V0b1 linearised with EcoRV. To construct pBG2-V1a-ins-tdtmt-ins and pBG2-V2a-ins-BFP-ins, each fragment of pCMV-tdTomato (Takara Bio) and pTagBFP2-N (Evrogen) was amplified by PCR with the following primers, F: 5′- GAACCTGCACTAGCCATCATGTTCTTTCCTGCGTTAT -3′, R: 5′- AAAAACGCGTGTCGATCCTGCACTAGCCATTTAAGATACATTGATGAGTT -3′. Then, each PCR product was digested with AflIII and ligated to a fragment of pinsB4ins prepared by HincII and AflII digestion. Then, fragments from pinsB4ins that contained tdTomato or TagBFP2-N were prepared by EcoRI digestion and ligated into pBG2-V1a for pBG2-V1a-ins-tdtmt-ins and pBG2-V2a for TagBFP2-N via MluI sites in each vector. To construct GLV2-EF1a-tdTomato, tDNA-pEF1a-BGHpA, a synthesised DNA, was digested by EcoRI and ligated in an annealed doubled-stranded palindrome oligo DNA, 5′- AATTCTGACTGTCTAGACAGTCA -3′, which included an XbaI site. Then, tDNA-pEF1a-BGHpA-XbaI was digested by XbaI and ligated to a fragment of pCMV-tdTomato digested by NheI and AvrII. tDNA-pEF1a-tdTomato was digested by AscI and NheI, and ligated to a PCR product amplified by PCR from pBG2-Vla using the following primers: BxbI-PhiC31_CL_F: 5′- CGCATGGCGCGCCTGGCCGTGGCCGTGCTCGTC -3′ and BxbI-PhiC31_CL_R: 5′- CTAGTCCTAGGGACCCTACGCCCCCAACTGA -3′ and by digestion with AscI and NheI. To construct GLV3-NeoR-pEF1a-BFP, tDNA-EF1a-BGHpA, a synthesised DNA was digested by EcoRI and ligated to a PCR product amplified from pTagBFP2-N by primers BFP_cl_EcoRI_F: 5′- CGAGTCGAATTCGCCACCATGGTGTCTAAGGGCGAAGAGCTGA -3′ and BFP_cl_EcoRI_R: 5′- TGTAACGAATTCCTATTAATTAAGTTTGTGCCCCAGTTTGC -3′, and digested by EcoRI. Furthermore, tDNA-EF1a-BFP was digested by AscI and NheI, and ligated to a PCR product amplified from pBG2-V2a by primers PhiC31 attB 3’HPRT Asc1 F: 5′- CGCATGGCGCGCCGATGTAGGTCACGGTCTCGAAG -3′ and PhiC31 attB 3'HPRT cl R: 5′- CTAGTCCTAGGAGGCTGGTTCTTTCCGCCT -3′, and digested by AscI and AvrII (GLV3-pEF1a-BFP). Then, GLV3-pEF1a-BFP was digested by AscI and Hind III, and ligated to three DNA fragments amplified by PCR from GLV3-BFP with primers AscI-PhiC31 attB-AvrII F: 5ʹ- GGCCGCATGGCGCGCCGATG -3′ and AscI-PhiC31 attB-AvrII R: 5ʹ- AAAACCTAGGTCATCATGATGGACCAGATG -3′, pBG2-V1b1 with primers AvrII-NeoR-AgeI F: 5′- AAAACCTAGGGCGGCCGCCGTGACCTGCAC -3′ and AvrII-NeoR-AgeI R: 5′- AAAAACCGGTCCCCAGCTGGTTCTTTCCGC -3′, and an annealed double-stranded oligomer DNA with primers AgeI-HindIII oligo F: 5′- AGCTTGATTTCGGCCTATTGA -3′ and AgeI-HindIII oligo R: 5′- CCGGTCAATAGGCCGAAATCA -3′. The mCherry expression vector with a Cre-loxP system was a modified X3.1-I-EGFP-I vector^48^. X3.1-I-EGFP-I that contained the Cre-loxP system was digested with NcoI and SpeI, which removed EGFP. The HS4 insulator on X3.1-I-EGFP-I was amplified with primers 5′- ATCCATGGATCGACTCTAGAGGGACAGCC -3′ and 5′- ATAACTAGTCGACGCGGCCGCCTCACTGACTCCGTCCTGGA -3′, and ligated using NcoI and SpeI sites. mCherry expression vectors with three types of constitutive promoters (PGK, EF1α, and CAG) were purchased from VectorBuilder (Chicago, IL, USA). The vector information is available from the database of VectorBuilder with the following vector IDs: pRP-[Exp]-hPGK > mCherry), pRP-[Exp]-EF1A > mCherry, and pRP-[Exp]-CAG > mCherry. These mCherry expression cassettes were prepared by NotI digestion of the mCherry expression vector. Then, each mCherry expression cassette was inserted into the modified X3.1 without EGFP.

### Transfection and gene loading into each HAC/MAC by Cre-loxP and SIM systems

HEK293 and HT1080 cells that contained HACs/MACs were prepared at 5 × 10^6^ cells per 10-cm dish. HEK293 and HT1080 cells were transfected using a previously described method^25^. Transfected plasmids were 3.5 µg pBG2-V0b-ins-ELuc-ins, 7 µg pBG2-V1a-ins-tdTomato-ins, 10.5 µg pBG2-V2a-ins-BFP-ins, 3 µg pBS185 (pCMV-Cre), 3 µg pCMV-Bxb1 integrase, and 3 µg pCMV-PhiC31 integrase. These plasmids were mixed and transfected with Lipofectamine 2000 (Invitrogen), following the manufacturer’s instructions. Then, the transfected cells were selected in 2% HAT medium. hiPSCs that contained HACs/MACs were prepared at 2 × 10^6^ cells per 10-cm dish. The introduced plasmids were 3.5 µg pBG2-V0b1-ins-ELuc-ins, 7 µg GLV2-tdTomato, 10.5 µg GLV3-NeoR-BFP, 3 µg pEF1a-Cre, 3 µg pCAG-Bxb1 integrase, and 3 µg pCAG-PhiC31 integrase. The mixture of plasmids was introduced into hiPSCs by a NEPA21 electroporator (NEPAGENE, Ichikawa, Japan) with the following conditions: pouring pulse, 135 or 175 V; pulse length, 2.5 milliseconds; pulse interval 50 ms; number of pulses, 2; decay rate, 10% and polarity + , and then transfer pulse, 20 V; pulse length, 50 milliseconds; pulse interval, 50 milliseconds, number of pulses, 2, decay rate, 40% and polarity, + /−. Then, the electroporated cells were expanded in the 10-cm dish and 90 µg/mL G418 was added to the culture medium at 48 h after electroporation. We also used another method of electroporation by a Nucleofector 4D (Lonza, Basel, Switzerland). The introduced plasmids were 3.5 µg pBG2-V0b-ins-ELuc-ins, 7 µg GLV2-tdTomato, 10.5 µg GLV3-NeoR-BFP, 3 µg pEF1a-Cre, 3 µg pEF1a-Bxb1 integrase, and 3 µg pEF1a-PhiC31 integrase. A total of 1 × 10^6^ hiPSCs and plasmids were mixed with P3 Primary Cell 4D-Nucleofector™ X Kit L (Lonza), following the manufacturer’s instructions, and pulsed with the CA-137 program. Eight micrograms of X3.1 that contained mCherry with each constitutive promoter and 2 µg of a Cre expression vector (pBS185) were mixed and introduced into hiMSCs by the NEPA21 electroporator with the following conditions: pouring pulse, 175 V; pulse length, 2.5 millisconds; pulse interval, 50 milliseconds; the number of pulses, 2; decay rate, 10% and polarity + , and then transfer pulse, 20 V; pulse length, 50 milliseconds, pulse interval, 50 milliseconds, number of pulses, 2; decay rate 40% and polarity + /−. Then, hiMSCs were selected in 2% HAT medium. pBS185 CMV-Cre was a gift from Brian Sauer (Addgene plasmid # 11,916; http://n2t.net/addgene:11916; RRID: Addgene_11916)^49^.

### Gene knockout by CRISPR/Cas9

Gene knockout of HPRT1 was performed in HEK293 cells, hiMSCs, and hiPSCs with the multiplex CRISPR/FokI-dCas9 vector system^50,51^. The multiplex CRISPR/FokI-dCas9 vector system targeted six sequences for knockout of the HPRT1 gene: HPRT T1: 5′- TAACGGAGCCGGCCGGCGCGCGG -3′, HPRT T2: 5′- TGGCGTCGTGGTGAGCAGCTCGG -3′, HPRT T3: 5′- AAATCCTCAGCATAATGATTAGG -3′, HPRT T4: 5′- CTCATGGACTAATTATGGACAGG -3′, HPRT T5: 5′- CACAGAGGGCTACAATGTGATGG -3′ and HPRT T6: 5′- TAAATTCTTTGCTGACCTGCTGG -3′. The vector system was transfected into HEK293 cells and then HPRT knockout cells were screened by 100 µM 6TG treatment. HT1080 clones with spontaneous mutation of the HPRT1 gene were selected by 6TG treatment. Gene knockout of the neomycin resistance gene was performed with CRISPR/Cas9 targeting 5ʹ- AGGCTATTCGGCTATGACTGGG -3′^27^.

### PCR analysis

PCR analyses were performed with KOD Fx (TOYOBO, Osaka, Japan), following the manufacturer’s instructions. The primers to detect gene insertion in HACs/MACs via the SIM system were HPRT junc sp F 5′- CGGCTTCCTCCTCCTGAACAA -3′ and HPRT junc sp R 5ʹ- TCCATAAGACAGAATGCTATGCAACC -3′ for HPRT EX1-2 and HPRT EX3-9 reconstitution in HEK293, HT1080 and hiMSCs, and TRANS L1 5ʹ- TGGAGGCCATAAACAAGAAGAC -3ʹ and SIM Neo Rv 5ʹ- CGCCTTGAGCCTGGCGAACA -3′ for HPRT EX1-2 and Neo cDNA reconstitution in human iPSCs.

### FISH analysis

Cells were treated with colcemid to induce metaphase arrest, treated with 0.075 M KCl, and then fixed with methanol/acetate (3:1) (FUJIFILM Wako). FISH was performed with the p11-4 alpha satellite probe^52^ to stain the alpha satellite of hChr.13, 21 and HAC, and mouse Cot-1 DNA to stain the MAC. The probes were labelled with digoxigenin (Roche, Basel, Schweiz) and the inserted plasmid vector targeted to the chromosome fragment was labelled with biotin (Roche). The DNA probes were labelled with a nick translation kit (Roche), following the manufacturer’s instructions. The detailed protocol has been described previously^26^.

### Teratoma formation and histological analysis

Mice were maintained under specific pathogen-free conditions with a 12-h light–dark cycle. Human iPSCs (1 × 10^6^) were subcutaneously transplanted into a testis of anaesthetised severe combined immunodeficiency mice (Charles River, Yokohama, Japan). A mixed anaesthetic agent prepared with 0.3 mg/kg medetomidine hydrochloride, 4 mg/kg midazolam, and 5 mg/kg butorphanol tartrate was administered intraperitoneally to the mice. Teratomas appeared after ~ 8 weeks. The anaesthetised mice were sacrificed and the teratomas were collected. Then, the teratomas were fixed with 20% neutral formalin/PBS and processed for paraffin sectioning. The sections were stained with haematoxylin and eosin.

### FCM analysis

To evaluate the ratio of cells that expressed fluorescent proteins, the cells were analysed by FCM using a BD LSRFortessa X-20 flow cytometer (Beckton Dickinson) equipped with 488, 405, and 561 nm lasers to detect EGFP, BFP2, and tdTomato, respectively. The ratio of fluorescence-positive cells of each fluorescent protein was determined by measuring 10,000 cells in each experiment.

### Luciferase assay

The assay was performed with 6 × 10^4^ cells in each well of a 96-well plate. Luciferase activity was measured with Emerald Luc Luciferase Assay Reagent Neo (TOYOBO), following the manufacturer’s instructions. Bioluminescence was measured for 1 s by an EnVision (PerkinElmer, Waltham, MA, USA)^26^.

### RT-PCR

Total RNA was isolated with a Nucleospin RNA plus kit (Takara Bio), following the manufacturer’s instructions. cDNA was synthesised with a PrimeScript™ II 1^st^ strand cDNA Synthesis Kit (Takara Bio), following the manufacturer’s instructions. RT-PCR analyses were performed with KOD one, cDNA, and the following primer sets: CXCL1 F 5′- TGTGAAGGCAGGGGAATGTA -3′ and R 5′- GCCCCTTTGTTCTAAGCCAG -3′, CD90 F 5′- ATGAACCTGGCCATCAGCA -3′ and R 5′- GTGTGCTCAGGCACCCC -3′, IL-8 F 5′- ACCGGAAGGAACCATCTCAC -3ʹ and R 5′- ATTTGGGGTGGAAAGGTTTG -3′, and CCL2 F 5′- GCAGCAAGTGTCCCAAAGAA -3′ and R 5′- AACAGGGTGTCTGGGGAAAG -3ʹ.

### Quantitative RT-PCR

qRT-PCR analysis was performed with TB Green® Fast qPCR Mix (Takara Bio), following the manufacturer’s instructions. The following primer sets were used: mCherry F 5′- AAGGGCGAGGAGGATAACAT -3′ and R 5′- ACATGAACTGAGGGGACAGG -3′, GAPDH1F 5′- AGCCACATCGCTCAGACAC -3′, R 5ʹ- GCCCAATACGACCAAATCC -3′. OCT 3/4 F 5′- AGAAGGATGTGGTCCGAGTGTG -3′, R 5′- CCACCCTTTGTGTTCCCAATTCC -3′. The enzyme reactions and measurements were performed with the Applied Biosystems 7300 Fast Real-Time PCR System (Thermo Fisher Scientific), following the manufacturer’s instructions. Fold changes in the gene expression level of mCherry were calculated using the ∆∆Ct method and normalised to GAPDH gene expression.

### Immunofluorescence staining

iPS cells were fixed with 4% paraformaldehyde/PBS (Sigma Aldrich) in a freezer overnight. The samples were blocked with PBS that contained 5% dry skim milk and 0.1% NP-40 (FUJIFILM Wako) for 15 min at RT. Then, the samples were stained for 1 h at RT with mouse IgG2b against OCT3/4 (1:50) (Santa Cruz Biotechnology, sc-5279, Dallas, TX, USA) as the primary antibody and then anti-mouse IgG2b-Alexa 555 (1:500) (Thermo Fisher Scientific, A-21147) for detection. Image were obtained under a Keyence BZ-X800 (Keyence, Osaka, Japan).

### Alkaline phosphatase staining

The iPS cells were prepared in confluently and fixed with 4% paraformaldehyde/PBS (Sigma Aldrich) in a freezer overnight. The staining was performed with Alkaline Phosphatase Staining Kit (Cosmo Bio Co., LTD., Tokyo, Japan). The image were obtained by microscopy for cell culture (OLYMPUS CORPORATION, Tokyo, Japan) and the attached CCD camera DP22 and Software.

## Supplementary Information

Supplementary Information.

## Data Availability

The datasets of vector sequences and cell lines generated during and/or analysed during the current study are available from the corresponding author on reasonable request.

## Abbreviations

HAC: Human artificial chromosome
MAC: Mouse artificial chromosome
MMCT: Microcell-mediated chromosome transfer
hiMSC: Human immortalised mesenchymal stem cell
hiPSC: Human induced pluripotent stem cell
Tc: Trans-chromosomic
SIM: Simultaneous or sequential insertion of multiple genes-loading vectors
HPRT: Hypoxanthine-guanine phosphoribosyltransferase
MI: Multiple integrase
CRISPR: Clustered regularly interspaced short palindromic repeats
Cas9: CRISPR-associated protein 9
MV: Measles virus
ELuc: Emerald luciferase
FCM: Flow cytometry
FISH: Fluorescence in situ hybridisation
6TG: 6-Thioguanine
PDL: Population doubling level
FBS: Fetal bovine serum
HAT: Hypoxanthine-aminopterin-thymidine
