# Supplementary material for: Panel of human cell lines with human/mouse artificial chromosomes
Source: Sci Rep. 2022 Feb 22;12:3009. doi: 10.1038/s41598-022-06814-3 (PMC8863800; doi:10.1038/s41598-022-06814-3)
Supplement: Supplementary file 1 — Supplementary Information. [file 41598_2022_6814_MOESM1_ESM.docx]

**Panel of human cell lines with human/mouse artificial chromosomes**

Narumi Uno^1,2,3,*^, Shuta Takata^1^, Shinya Komoto^1^, Hitomaru Miyamoto^1^, Yuji Nakayama^4^, Mitsuhiko Osaki^2,5^, Ryota Mayuzumi^3^, Natsumi Miyazaki^3^, Chiaki Hando^3^, Satoshi Abe^2^, Tetsushi Sakuma^6^, Takashi Yamamoto^6^, Teruhiko Suzuki^7^, Yoshihiro Nakajima^8^, Mitsuo Oshimura^2^, Kazuma Tomizuka^3^, Yasuhiro Kazuki^1,2,**^

1. Division of Genome and Cellular Functions, Department of Molecular and Cellular Biology, School of Life Science, Faculty of Medicine, Tottori University, 86 Nishi-cho, Yonago, Tottori 683-8503, Japan

2. Chromosome Engineering Research Center, Tottori University, 86 Nishi-cho, Yonago, Tottori 683-8503, Japan

3. Laboratory of Bioengineering, Faculty of Life sciences, Tokyo University of Pharmacy and Life Sciences, 1432-1 Horinouchi, Hachiohji, Tokyo 192-0392, Japan

4. Division of Radioisotope Science, Research Initiative Center, Organization for Research Initiative and Promotion, Tottori University, 86 Nishi-cho, Yonago, Tottori, 683-8503, Japan.

5. Division of Experimental Pathology, Department of Biomedical Sciences, Tottori University Faculty of Medicine, Yonago, Tottori 683-8503, Japan

6. Division of Integrated Sciences for Life, Graduate School of Integrated Sciences for Life, Hiroshima University, Higashi-Hiroshima, Hiroshima 739-8526, Japan

7. Stem Cell Project, Tokyo Metropolitan Institute of Medical Science, Kamikitazawa, Setagaya-ku, Tokyo 156-8506, Japan

8. Health Research Institute, National Institute of Advanced Industrial Science and Technology (AIST), Takamatsu, Kagawa 761-0395, Japan

*Corresponding author: narumi@toyaku.ac.jp, Tel: +81-42-676-7141

**Corresponding author: kazuki@tottori-u.ac.jp, Tel: +81-859-38-6219

**Supplemental Figure 1**


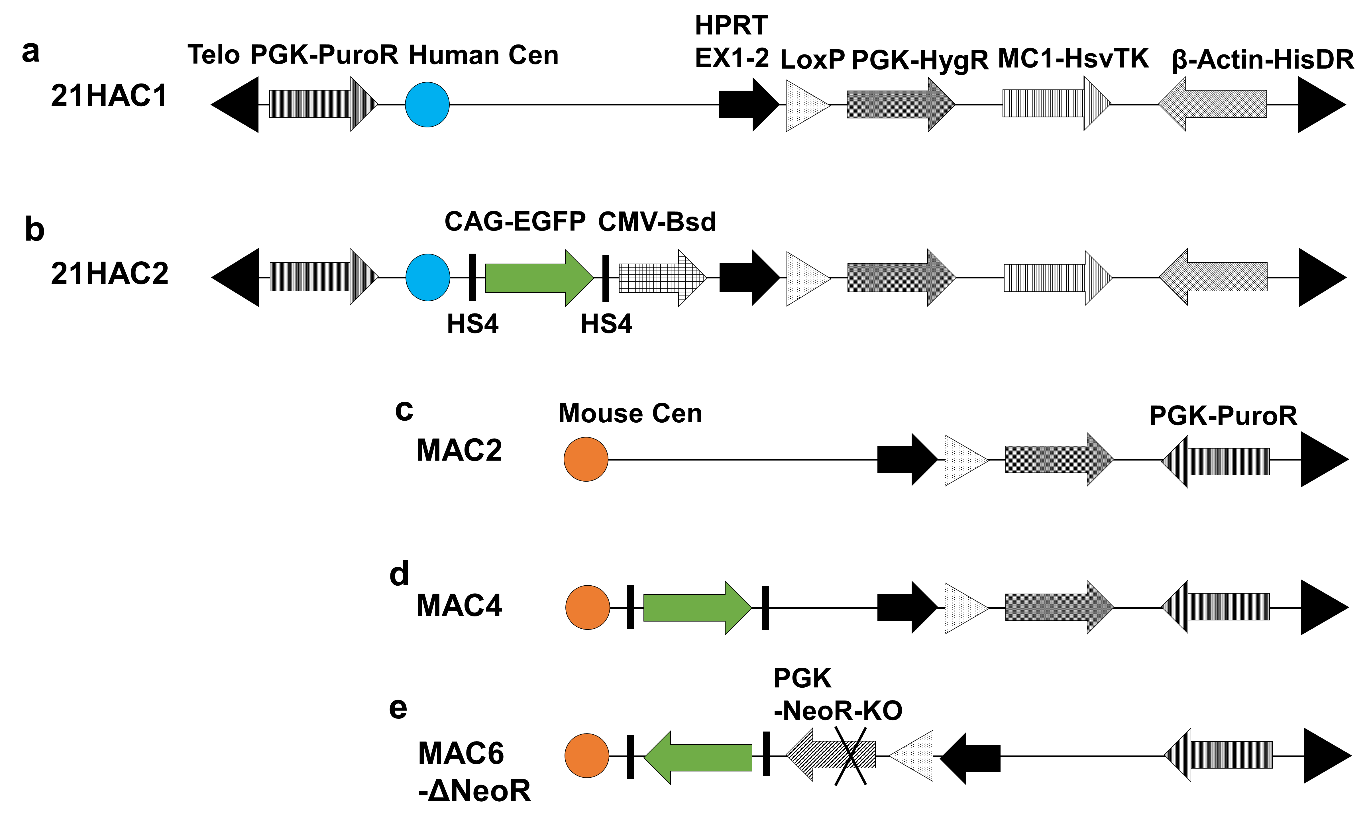


**Supplementary Figure. S1 Structure of HACs/MACs**

The features of HACs/MACs in terms of antibiotic resistance genes, fluorescent markers, and gene-loading sites are shown. (a) 21HAC1, (b) 21HAC2, (c) MAC2, (d) MAC4, and (e) MAC6-ΔNeoR.

**Supplemental Figure 2**


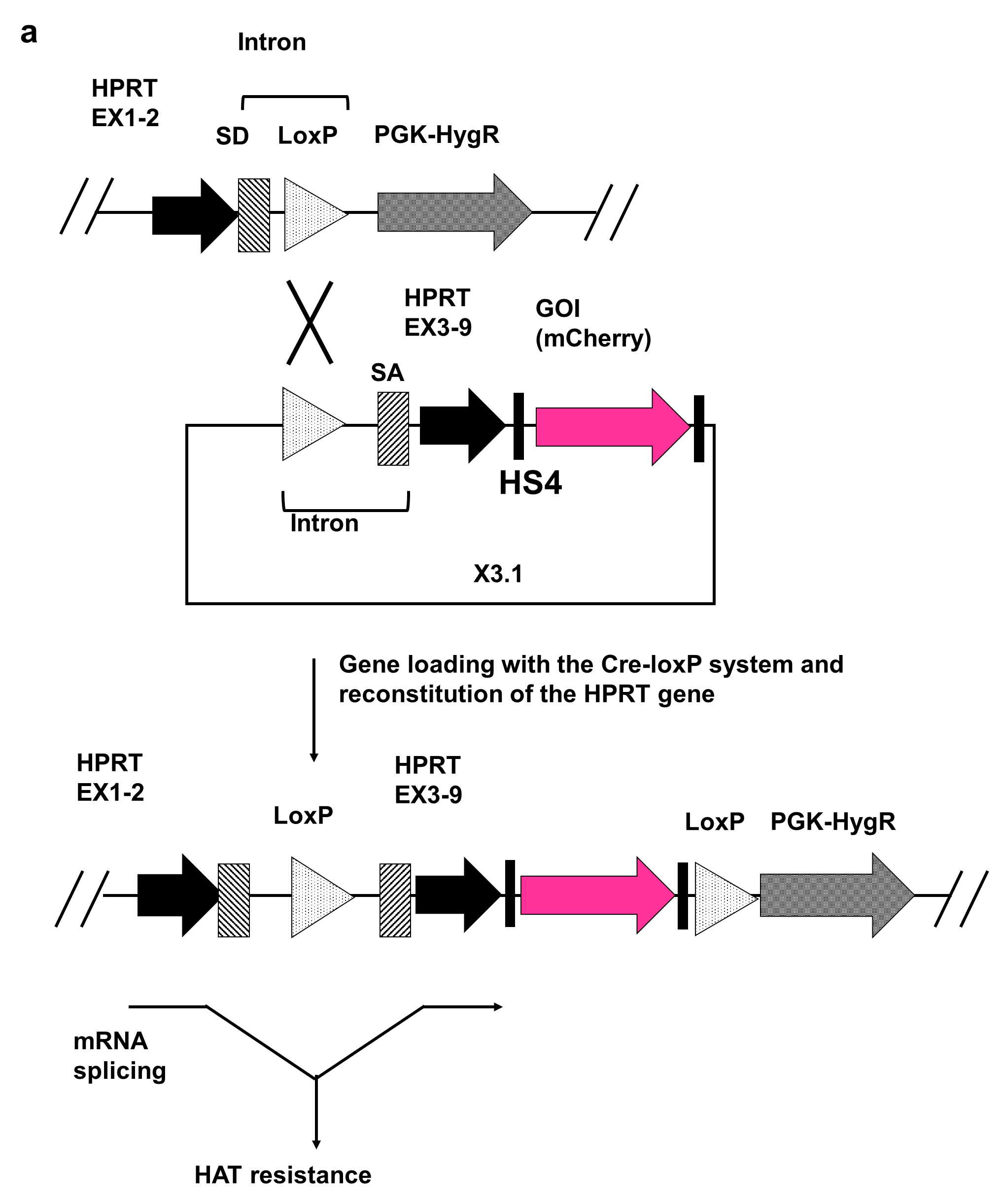


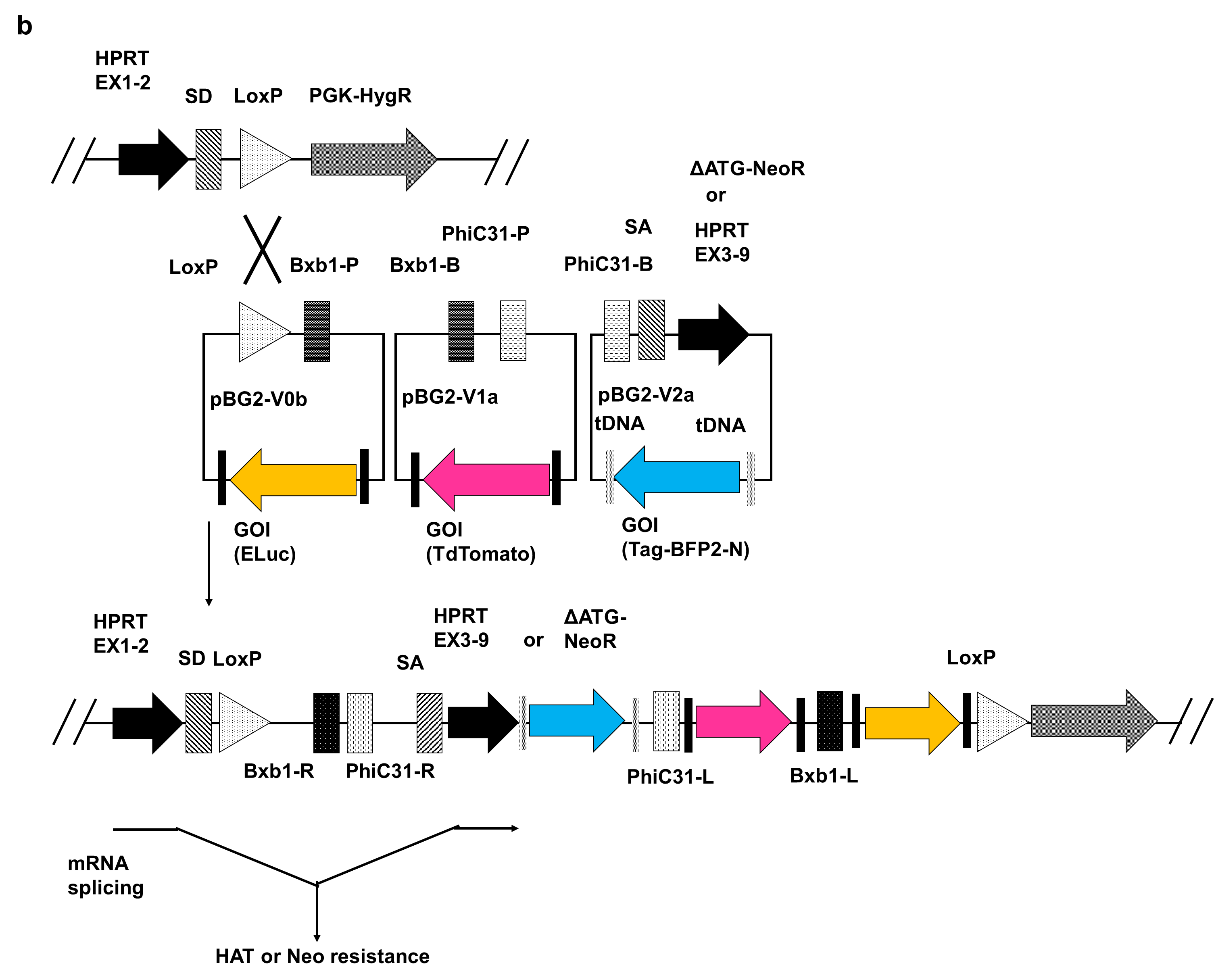


**Supplementary Figure S2 Schematic diagrams of gene-loading systems**

The gene-loading system using Cre-loxP and reconstitution of the HPRT1 gene is shown. The two types of gene-loading systems for HACs/MACs commonly contain exons 1 and 2 of the HPRT1 gene. The front half of the intron between exons 2 and 3, which includes a splicing donor site, and a loxP site are shown. The inserted plasmid vector(s) with base(s) X3.1 or pBG-V2a contain(s) the back half of the intron, which includes a splicing acceptor site, and exons 3–9 of the HPRT1 gene. Cells with inserted plasmid vector(s) acquire a complete HPRT1 gene and resistance against cell death in HAT medium, because the HPRT gene functions only when exons 1–9 align. (a) Insertion diagram of a plasmid vector with the Cre-loxP system that can insert a circular vector into HACs/MACs by cotransfection of X3.1 and Cre recombinase expression vectors. The gene of interest (GOI) is flanked by HS4 insulators for stable gene expression. (b) Schematic diagram of insertion of the three plasmid vectors with the SIM system that can insert three plasmid vectors simultaneously into HACs/MACs by cotransfection of the three types of pBG2 vectors and three plasmid vectors that express Cre recombinase, Bxb1 integrase, and PhiC31 integrase. Their enzymes recognise each specific sequence on the three pBG vectors and appropriate recombination, as shown in the figure, results in rebuilding of the HPRT1 gene, and cells with inserted plasmid vectors acquire HAT resistance. After inserting a vector that replaces the HPRT gene of pBG2-V2a with a Neo gene that lacks the first ATG, a fusion gene between HPRT1 and Neo genes is expressed and the cells acquire Neo resistance. Each GOI in pBG2-V0b and pBG2-V1a vectors is flanked by HS4 insulators and the GOI in pBG2-V2a is flanked by tDNA insulators.

**Supplemental Figure 3**


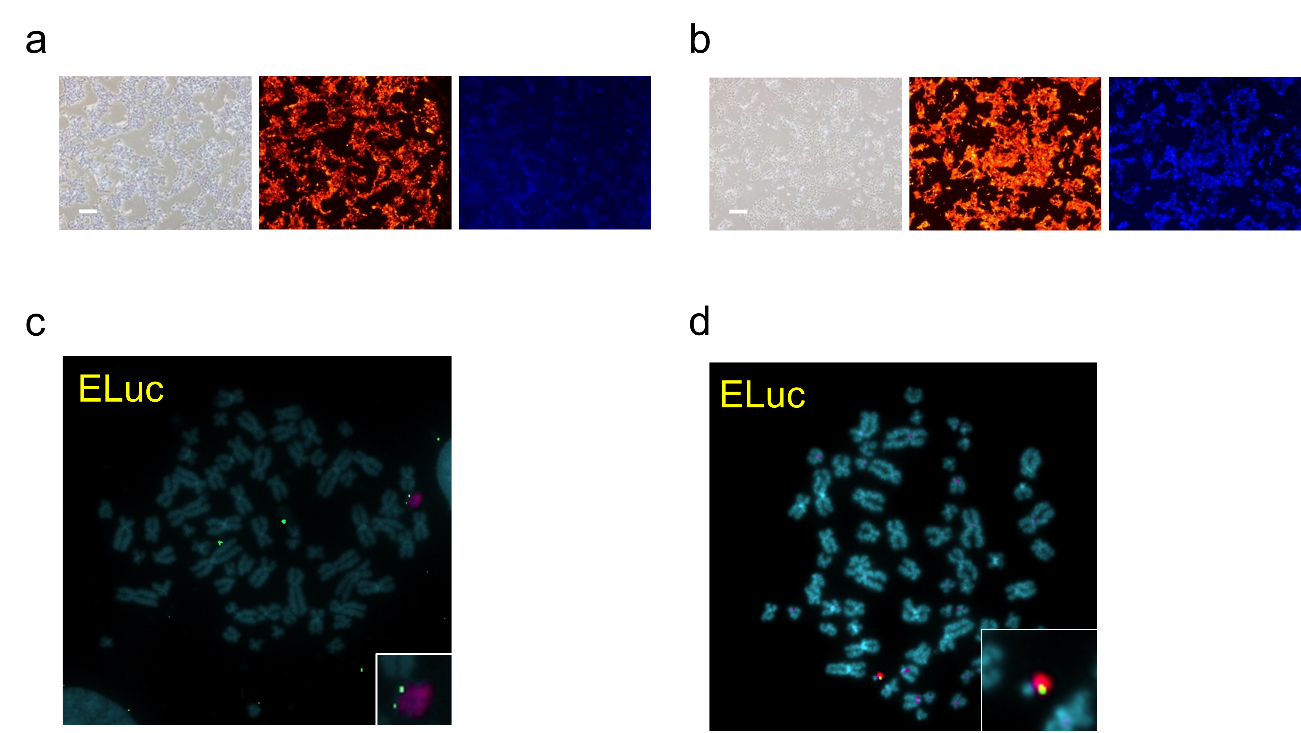


**Supplementary Figure S3 Analyses of HEK293 cells that contained MAC2 or 21HAC1s harbouring tdTomato, BFP, and ELuc via the SIM system**

(a) Representative images of fluorescent protein expression in HEK293 cells that contained MAC2 inserted with tdTomato, BFP, and ELuc via the SIM system. Brightfield (left), tdTomato (center), and BFP (right). Bar = 200 µm. (b) Representative image of fluorescent protein expression in HEK293 cells that contained MAC2 inserted with tdTomato, BFP, and ELuc via the SIM system. Bar = 200 µm. (c) Representative image of FISH analysis of HEK293 cells that contained 21HAC1 and the three plasmids. Red: alpha satellite probe (p11-4) staining the centromere of Chr.13, 21 and 21HAC1; green: pBG2-V0b-ins-ELuc-ins. (d) Representative image of FISH analysis of HEK293 cells that contained 21HAC1 and the three plasmids. Green: pBG2-V0b-ins-ELuc-ins.

**Supplementary Figure S4**


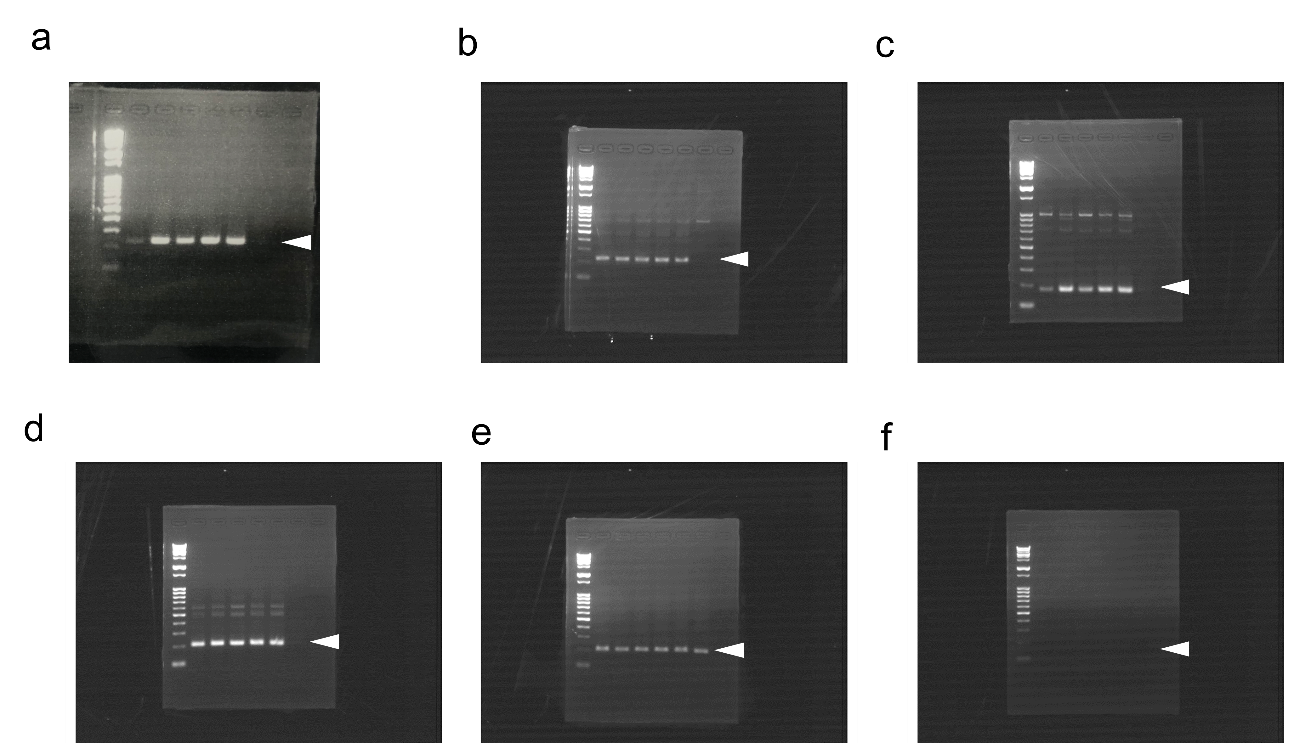


**Supplementary Figure S4 RT-PCR analysis of various MSC markers in hiMSCs that contained 21HAC2.**

The original gel-image of the results of RT-PCR analyses. (a) CXCL1, (b) CD90, (c) IL-8, (d) CCL2, (e) GAPDH2 RT+ and (f) GAPDH2 RT-. For all gels, the samples were applied as follows; i.e., Lane 1: Gene Ladder Wide 2 (NIPPON GENE CO., LTD. Tokyo. Japan.), lane 2: hiMSC, lane 3: hiMSC6TGr #3, lane 4: hiMSC6TGr #6, lane 5: hiMSC 21HAC2 A03, lane 6: hiMSC 21HAC2 D11 and lane 7: HEK293T. The allowheads shows the expected band of each analysis. For each experiment, the expected band was indicated by each arrowhead.

| **Cell line** | **Vector** | **Number of analyzed clones** | **Number of PCR-positive clones** |
| --- | --- | --- | --- |
| HEK293 | HAC1 | 6 | 5 |
| HEK293 | HAC2 | 3 | 3 |
| HEK293 | MAC2 | 18 | 15 |
| HEK293 | MAC4 | 14 | 13 |
| HT1080 | HAC2 | 15 | 13 |
| HT1080 | MAC4 | 13 | 12 |

**Supplemental Table S1**

**Supplementary Table S1 Numbers of analyzed clones and PCR-positive clones with correct insertion of the plasmids by the SIM system.**

The results for HEK293 and HT1080 cells are described in accordance with the maintained HAC/MAC.
